# Supplementary material for: Healthcare Service Utilisation Across Continuum of Care for Type 2 Diabetes Among Culturally and Linguistically Diverse Populations: A Systematic Review
Source: Int J Environ Res Public Health. 2025 Aug 15;22(8):1279. doi: 10.3390/ijerph22081279 (PMC12386074; doi:10.3390/ijerph22081279)
Supplement: Supplementary file 1 [file ijerph-22-01279-s001.zip › ijerph-3746806-supplementary.pdf]

**Table S1.** Mixed Methods Appraisal Tool (MMAT), version 2018.

| Category of study designs | Methodological quality criteria | Responses | | | |
|---|---|---|---|---|---|
| | | Yes | No | Can't tell | Comments |
| Screening questions (for all types) | S1. Are there clear research questions? | | | | |
| | S2. Do the collected data allow to address the research questions? | | | | |
| 1. Qualitative | 1.1. Is the qualitative approach appropriate to answer the research question? | | | | |
| | 1.2. Are the qualitative data collection methods adequate to address the research question? | | | | |
| | 1.3. Are the findings adequately derived from the data? | | | | |
| | 1.4. Is the interpretation of results sufficiently substantiated by data? | | | | |
| | 1.5. Is there coherence between qualitative data sources, collection, analysis and interpretation? | | | | |
| 2. Quantitative randomized controlled trials | 2.1. Is randomization appropriately performed? | | | | |
| | 2.2. Are the groups comparable at baseline? | | | | |
| | 2.3. Are there complete outcome data? | | | | |
| | 2.4. Are outcome assessors blinded to the intervention provided? | | | | |
| | 2.5 Did the participants adhere to the assigned intervention? | | | | |
| 3. Quantitative non-randomized | 3.1. Are the participants representative of the target population? | | | | |
| | 3.2. Are measurements appropriate regarding both the outcome and intervention (or exposure)? | | | | |
| | 3.3. Are there complete outcome data? | | | | |
| | 3.4. Are the confounders accounted for in the design and analysis? | | | | |
| | 3.5. During the study period, is the intervention administered (or exposure occurred) as intended? | | | | |
| 4. Quantitative descriptive | 4.1. Is the sampling strategy relevant to address the research question? | | | | |
| | 4.2. Is the sample representative of the target population? | | | | |
| | 4.3. Are the measurements appropriate? | | | | |
| | 4.4. Is the risk of nonresponse bias low? | | | | |
| | 4.5. Is the statistical analysis appropriate to answer the research question? | | | | |
| 5. Mixed methods | 5.1. Is there an adequate rationale for using a mixed methods design to address the research question? | | | | |
| | 5.2. Are the different components of the study effectively integrated to answer the research question? | | | | |
| | 5.3. Are the outputs of the integration of qualitative and quantitative components adequately interpreted? | | | | |
| | 5.4. Are divergences and inconsistencies between quantitative and qualitative results adequately addressed? | | | | |
| | 5.5. Do the different components of the study adhere to the quality criteria of each tradition of the methods involved? | | | | |

**Table S2.** Results of quality assessment of the included studies.

| Included studies | Domain | Score | | | | |
|---|---|---|---|---|---|---|
| | | Q1 | Q2 | Q3 | Q4 | Q5 |
| Alzubaidi et al, 2015 | Qualitative | 1 | 1 | 1 | 1 | 1 |
| Nam et al, 2013 | Qualitative | 1 | 1 | 1 | 1 | 1 |
| Renfrew et al, 2013 | Qualitative | 1 | 1 | 1 | 1 | 1 |
| Park et al, 2023 | Qualitative | 1 | 1 | 1 | 1 | 1 |
| Gele et al, 2015 | Qualitative | 1 | 1 | 1 | 1 | 1 |
| Hu et al, 2013 | Qualitative | 1 | 1 | 1 | 1 | 1 |
| Leung et al, 2014 | Qualitative | 1 | 1 | 1 | 1 | 1 |
| Almansour et al, 2017 | Qualitative | 1 | 1 | 1 | 1 | 1 |
| Biyikli et al, 2017 | Qualitative | 1 | 1 | 1 | 1 | 1 |
| Barbara et al, 2013 | Qualitative | 1 | 1 | 1 | 1 | 1 |
| Rose et al, 2015 | Qualitative | 1 | 1 | 1 | 1 | 1 |
| Kokanovic et al., 2006 | Qualitative | 1 | CT | 1 | 1 | 1 |
| Choi et al, 2018 | Qualitative | 1 | 1 | 1 | 1 | 1 |
| Fagerli et al, 2005 | Qualitative | 1 | CT | CT | 1 | CT |
| Rhodes et al, 2003 | Qualitative | 1 | 1 | 1 | 1 | 1 |
| Heisler et al, 2009 | Qualitative | 1 | 1 | 1 | 0.5 | 0.5 |
| Cokluk et al, 2023 | Qualitative | 1 | 1 | 1 | 1 | 1 |
| Moore et al, 2022 | Qualitative | 1 | 1 | 1 | 1 | 1 |
| Ho et al, 2006 | Qualitative | 1 | 1 | 1 | 1 | 1 |
| Jager et al, 2020 | Qualitative | 1 | 1 | 1 | 1 | 1 |
| Omodara et al, 2022 | Qualitative | 1 | 1 | 1 | 1 | 1 |
| Ramal et al, 2012 | Qualitative | 1 | 1 | 1 | 1 | CT |
| Patel et al, 2023 | Qualitative | 1 | 1 | 1 | 1 | 1 |
| Utz et al, 2006 | Qualitative | 1 | 0 | 1 | 1 | 0 |
| Cha et al, 2012 | Qualitative | 1 | CT | 1 | 1 | 1 |
| Lipton et al, 1998 | Qualitative | 1 | CT | 1 | CT | CT |
| Mary et al, 2013 | Qualitative | 1 | CT | 1 | 1 | 1 |
| Kokanovic et al., 2007 | Qualitative | 1 | 1 | 1 | 1 | 1 |
| van Allen et al, 2021 | Qualitative | 1 | 1 | 1 | 1 | 1 |
| Joo et al, 2016 | Qualitative | 1 | 1 | 1 | 1 | CT |
| Carolan-Olah et al, 2018 | Qualitative | 1 | 1 | 1 | 1 | 1 |
| Kollannoor-Samuel et al, 2012 | Quantitative | 1 | 0 | 1 | CT | 1 |
| Hyman et al, 2012 | Quantitative | 1 | CT | 1 | CT | 1 |
| Lu et al, 2016 | Quantitative | 1 | 0 | CT | CT | 1 |
| Lyles et al 2022 | Mixed methods | 1 | 1 | 1 | 1 | 1 |
